# Infective Endocarditis by *Pasteurella* Species: A Systematic Review

**DOI:** 10.3390/jcm11175037

**Published:** 2022-08-27

**Authors:** Angeliki Alifragki, Argyro Kontogianni, Ioanna Protopapa, Stella Baliou, Petros Ioannou

**Affiliations:** 1 School of Medicine, University of Crete, 70013 Voutes, Greece; 2 Internal Medicine Department, University Hospital of Heraklion, 71500 Voutes, Greece

**Keywords:** endocarditis, systematic review, *Pasteurella*

## Abstract

*Pasteurella* spp. are non-motile, facultative anaerobic, Gram-negative coccobacilli that are commonly found in the oral cavity and the gastrointestinal tract of some animals and are known to be the cause of infections. Usually, infections by *Pasteurella* spp. in humans is more common in the context of an animal bite leading to a skin and soft tissue infection (SSTI). Infective endocarditis (IE) is rarely caused by *Pasteurella* spp.; however, it can pose diagnostic and therapeutic dilemmas due to its rarity. The aim of the present study was to systematically review all cases of IE by *Pasteurella* spp. in the literature. A systematic review was performed of PubMed, Scopus and the Cochrane Library (through 20 December 2021) for studies providing data on epidemiology and clinical and microbiological characteristics as well as data on treatment and outcomes of IE by *Pasteurella* spp. A total of 28 studies containing data for 28 patients were included. Prosthetic valve was present in 21.4% of patients. The aorta was the most commonly involved intracardiac site. Fever, sepsis, septic shock and heart failure were the most common clinical presentations. Cephalosporins, aminopenicillins and penicillin were the antimicrobials used most commonly. Overall mortality was 17.9%.

## 1. Introduction

*Pasteurella* spp. belongs to the family *Pasteurellaceae* which also includes *Haemophilus*, *Actinobacillus*, *Mannheimia* and *Aggregatibacter*, among others [1,2]. *Pasteurella* spp. are non-motile, facultative anaerobic, Gram-negative coccobacilli with a length of 1 to 2 μm. They are able to grow in many commercially available culture media including sheep and chocolate agar but usually not in MacConkey agar media; additionally, most strains are catalase indole and oxidase positive. They are commonly found in the oral cavity and the gastrointestinal tract of some animals and are known to be the cause of infections such as septicemia [1]. More specifically, infections by *Pasteurella* spp. in humans are more common in the context of an animal bite leading to a skin and soft tissue infection (SSTI) [1]. *P. multocida* that was isolated for the first time after a cat bite in 1930 is the most clinically relevant microorganism of this genus. However, the first isolation of *Pasteurella* spp. was performed in 1878 from birds with cholera [1]. *P. multocida* appear as iridescent, smooth, blue colonies on common growth media, although encapsulated isolates may appear as mucoid. Five different serogroups (A–F) based on capsular antigens and 16 different serovars (1–16) based on somatic antigens have helped towards discrimination among strain differences of *P. multocida* with some of them being more frequently isolated from infected humans [3]. Currently, molecular techniques such as 16S-rRNA are more frequently used to discriminate between different *Pasteurella* spp. than serotyping [4,5].

Infective endocarditis (IE) is a rare infection that is associated with significant mortality and morbidity [6,7]. In a study that was conducted recently, in-hospital mortality for patients with this infection was 17% [8]. Data from another study in the United Kingdom showed that 30-day and 1-year mortality in patients with IE were 14% and 30%, respectively [9]. In cases of IE, the most commonly isolated microorganisms are Gram-positive, such as Staphylococci, Streptococci and Enterococci, which add up to 75% of isolated microorganisms in these infections [10,11]. However, even though the proportion may be low, there are several cases of IE caused by Gram-negative bacteria, most commonly from *Escherichia coli* and *Pseudomonas aeruginosa* [6,7,11]. Even though IE by Gram-negative microorganisms is uncommon, it can be associated with several diagnostic and therapeutic dilemmas due to clinicians’ lack of experience with this condition and the associated lack of published data and guidelines on its treatment [6]. Toward that direction, studies that provide information on IE by Gram-negative species would be of great value. Importantly, a review summarizing all current data on IE by *Pasteurella* spp. is lacking, with the exception of some case reports with literature review [12].

This study aimed to systematically review all cases of IE by *Pasteurella* spp. and describe the epidemiology, microbiology, clinical characteristics, treatment and outcomes.

## 2. Materials and Methods

### 2.1. Data Search

For this review, we adopted the Meta-analysis of Observational Studies in Epidemiology (MOOSE) guidelines that are more appropriate for systematic reviews assessing epidemiological studies [13]. Eligible studies were identified through searches of PubMed, Scopus and the Cochrane Library with the following key words: *Pasteurella* AND endocarditis. The day of last search was 20th December 2021.

### 2.2. Study Selection

Studies were included in the analysis if they met the following criteria: (1) published in English and (2) reporting data on patients’ clinical characteristics, microbiology, treatment and outcomes. Studies with the following criteria were excluded from the analysis: (1) secondary research papers (e.g., reviews), editorials and papers not reporting results on primary research; (2) studies not in humans; (3) studies not in English and (4) studies not referring to IE by *Pasteurella* spp. Two investigators (AK, IP) using Abstrackr [14] independently reviewed the titles and abstracts of the resulting references; then, they retrieved and rescreened the full-text publications of potentially relevant articles. Study selection was based on consensus. Reference lists of included studies were searched for relevant articles. When the investigators were unable to access a full-text publication, attempts were made to communicate with the study authors in order to kindly provide the full text.

### 2.3. Outcomes of Interest

The primary outcomes of the study were recorded data on the following: (a) epidemiology of patients with IE by *Pasteurella* spp. and (b) patients’ outcomes. Secondary outcomes were recorded data on (a) the exact site of infection, (b) the patients’ clinical characteristics, (c) their antimicrobial susceptibility and d) their treatment.

### 2.4. Data Extraction and Definitions

Data from each eligible study were extracted by three investigators (AA, AK, IP). The extracted data included study type, year of publication and country; patient demographic data (age and gender); patients’ relevant medical history (previous cardiac surgery or cardiac valve replacement, time after cardiac valve replacement); infection data and microbiology (infection site, isolated strains, site of microorganism isolation, presence of complications, presence of embolic phenomena); treatment administered for IE and outcomes (i.e., cure or death). Data on microbiology and association of mortality with the index infection were reported by the study authors. The diagnosis of IE was confirmed by the investigators based on information provided by the authors and the modified Dukes’ criteria if the diagnosis was at least possible (at least 1 major and 1 minor criterion or at least 3 minor criteria) or if pathological data established a diagnosis of IE [15]. The recorded complications included any organ dysfunction or clinical deterioration that was considered by the authors to be related to IE. The quality of evidence of included studies’ outcomes was assessed using the Grading of Recommendations Assessment, Development and Evaluation (GRADE) [16].

### 2.5. Statistical Analysis

Data are presented as number (%) for categorical variables and median (interquartile range, IQR) or mean (+/− standard deviation, SD) for continuous variables. The abovementioned statistics were calculated with GraphPad Prism 6.0 (GraphPad Software, Inc., San Diego, CA, USA).

## 3. Results

### 3.1. Literature Search

**Figure 1:**
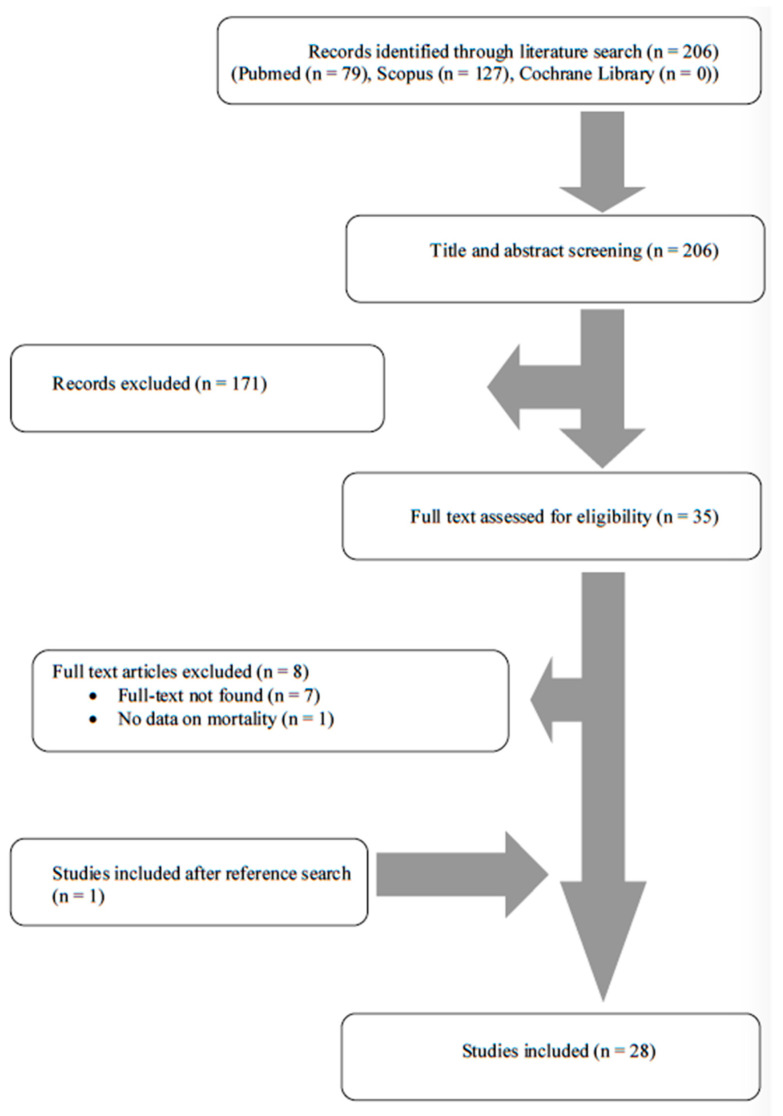
Flow diagram of study inclusion.

A total of 206 articles from PubMed, Scopus and the Cochrane Library were screened. After the authors reviewed the titles and abstracts, 35 articles were selected for full-text review. From these studies, 8 were excluded from the review: 7 articles could not be found, and 1 article did not present data on mortality. Additionally, 1 study was included after reference search of the aforementioned studies. Finally, 28 met the present study’s inclusion criteria [12,17,18,19,20,21,22,23,24,25,26,27,28,29,30,31,32,33,34,35,36,37,38,39,40,41,42,43]. The review process is graphically presented in Figure 1.

### 3.2. Included Studies’ Characteristics

The 28 studies that were finally included in the present analysis involved 28 patients in total. Supplementary Table S1 summarizes the characteristics of the included studies. Among those studies, 11 were conducted in North and South America, 10 in Europe and 7 in Asia. There were 28 case reports; thus, the overall quality of the evidence that contributed to this systematic review was rated as very low [16].

### 3.3. Epidemiology of IE by Pasteurella Spp.

The ages of patients with IE by *Pasteurella* spp. ranged from 21 to 88 years, the mean age was 56.4 years and 71.4% of patients (20 out of 28 patients) were male. A prosthetic cardiac valve was present in 21.4% (6 out of 28 patients) and was bioprosthetic in 50% of the cases (3 patients). Importantly, an animal bite or close contact with an animal was noted in the history in 75% (21 out of 28 patients). More specifically, in 21.4% (6 patients), an animal bite was noted in the recent history, and in 3.6% (1 patient), an animal scratch was noted in the recent history. Only one patient had sought medical attention and was treated for the animal bite. Animal contact involved cats in 65% (13 out of 20 patients with available data), dogs in 50% (10 patients) and sheep in 5% (1 patient). The median duration between animal bite and presentation due to infection was 11.5 days (range: 8–14 days). Among patients with IE by *Pasteurella* spp., 10.7% (3 patients) had liver cirrhosis, and 3.6% (1 patient) had immunosuppression as he was a kidney transplant recipient. Table 1 shows the characteristics of patients with IE by *Pasteurella* spp.

### 3.4. Microbiology of IE by Pasteurella Spp.

All patients had positive blood cultures for *Pasteurella* spp. The median number of positive blood cultures was 4 (range 1–10). Isolated species included *P. multocida* in 71.4% of patients (20 out of 28), *P*. *dagmatis* in 10.7% (3 patients), *P. pneumotropica* in 7.1% (2 patients), *P. haemolytica* in 7.1% (2 patients) and *P. aerogenes* in 3.6% (1 patient). Infection was polymicrobial in 7.1% (2 patients).

Antimicrobial susceptibility data were not provided in all studies; however, when data were available, *Pasteurella* spp. were susceptible to penicillin, ampicillin, cephalosporins, aminoglycosides and tetracyclines. The resistance rates to these antimicrobials were 0% (0 out of 11 cases with available data) for penicillin, 0% (0 out of 8 cases) for ampicillin, 0% (0 out of 8 cases) for cephalosporins, 11.1% (1 out of 9 cases) for aminoglycosides and 0% (0 out of 7 cases) for tetracyclines.

### 3.5. Diagnosis of IE by Pasteurella Spp.

The most common intracardiac site of infection was the aortic valve in 50% (13 out of 26) of patients and the mitral valve in 30.8% (8 patients). In 3.8% (1 patient), multiple valves were infected. Diagnosis was facilitated by transthoracic echocardiography in 48% (12 out of 25 patients) and by transesophageal echocardiography in 28% (7 patients), while diagnosis was set at autopsy in 10.7% (3 out of 28 patients) and with valve culture and histology in 28% (7 out of 25 patients). In 3.6% (1 out of 28 patients), diagnosis was made on empirical grounds due to lack of data from echocardiography. However, in all cases, diagnosis was confirmed with the current modified Dukes’ diagnostic criteria by this study’s investigators.

Regarding echocardiography, in 88.9% (24 out of 27 patients with available data) at least 1 vegetation was noted, with data regarding the vegetation size being provided by nine studies. The median length of vegetation was 1.1cm (range: 0.57cm–3.2cm). A paravalvular abscess was present in 14.8% (4 out of 27 patients), and a paravalvular leak was noted in 3.7% (1 out of 27 patients).

### 3.6. Clinical Characteristics of IE by Pasteurella Spp.

Durations of symptoms varied, with the median duration being 20 days (IQR: 5.5 to 30 days). Fever was present in 96.3% of patients (26 out of 27 patients with available data), sepsis in 81.5% (22 out of 27 patients), septic shock in 21.4% (6 out of 28 patients), heart failure in 21.4% (6 patients), embolic phenomena in 14.3% (4 patients) and immunologic phenomena in 17.9% (5 patients). Immunological phenomena included petechiae in one patient, glomerulonephritis in one patient, glomerulonephritis and petechiae in one patient, Osler nodules in one patient and arthritis in another patient. Finally, 14.8% (4 patients) developed a paravalvular abscess.

### 3.7. Treatment and Outcomes of IE by Pasteurella Spp.

The treatments provided for IE by *Pasteurella* spp. can be seen in detail in Supplementary Table S1 and in summary in Table 1. The duration of treatment among survivors ranged from 2 to 15 weeks, with a median duration of 6 weeks. Patients treated with an antimicrobial regimen including an aminoglycoside amounted to 22.2% (6 out of 27 with available data). Mortality among them was 50% (3 patients) and was higher than the mortality among patients treated without an aminoglycoside, 9.5% (2 out of 21 patients). Surgical management along with antimicrobials was performed in 42.9% (12 out of 28 patients). More specifically, five patients underwent aortic valve replacement; three patients underwent mitral valve replacement; one patient underwent pulmonary valve replacement; one patient underwent mitral valve repair; one patient underwent aortic valve replacement and aorta ascendens replacement; and one patient underwent atrial perforation repair, Bentall procedure and tricuspid valve replacement. Overall mortality was 17.9% (5 out of 28 patients) and was attributed directly to IE in all those cases.

Table 2 shows a comparison of patients’ characteristics according to whether they survived or died from the infection. Patient subgroups had similar characteristics; however, patients who survived had a trend towards a higher rate of a history of prosthetic valve, a lower rate of presentation with septic shock and embolic phenomena, a lower rate of aminoglycoside use and a higher rate of having had surgery along with antimicrobial treatment, even though the low numbers of patients precluded statistical analysis. In terms of antimicrobial treatment, all patients were treated with a beta-lactam antibiotic, while in 60% of patients who died (3 out of 5 patients), an aminoglycoside was used in combination.

## 4. Discussion

The present study described the characteristics of patients who developed IE by *Pasteurella* spp. The intracardiac site that is most commonly involved was the aortic valve. The most common clinical presentation included fever, sepsis, septic shock and heart failure. Cephalosporins, aminopenicillins and penicillin were the antimicrobials that were used most frequently, and 17.9% of patients died.

Infections by *Pasteurella* spp., more specifically by *P. multocida*, seem to have a worldwide distribution based on published series of infected patients. The basic reservoir for most *Pasteurella* spp. is in animals [1]. For example, dogs and cats have very high colonization rates, with colonization being asymptomatic in the vast majority of cases; however, respiratory tract infections and septicemia have been described in animals [2,44]. In humans, colonization by *Pasteurella* spp. may occur mostly in patients with underlying upper or lower respiratory diseases, such as chronic obstructive pulmonary disease (COPD), bronchiectasis and chronic sinusitis [45,46]. Human infection by *Pasteurella* spp. can be considered to be associated with an animal bite, associated with animal exposure other than an animal bite or occuring without evidence of exposure to animals. The most common type of infection by *Pasteurella* spp. is the one associated with animal bite [47,48,49]. For example, *Pasteurella* spp. are the most commonly isolated pathogens after infection associated with dog and cat bites, being present in 50% and 75% of infections, respectively [49,50,51,52,53,54]. Cat bites seem to be the most common cause of such infections, probably due to higher colonization of cats by *Pasteurella* spp., followed by dogs and other animals [49,51]. In the present study, animal contact without a history of a bite was the most common epidemiological factor, followed by a history of an animal bite. In the vast majority of patients, animal contact involved cats.

Human infections by *Pasteurella* spp. are also known to occur in patients who do not report a history of animal bite but do report close contact with animals. Persons at risk include veterinarians, pet owners, food handlers and farmers, and examples of such infections may include SSTIs, pneumonia, bone and joint infections, meningitis and endocarditis [47,55,56]. Finally, a significant percentage of infections caused by *Pasteurella* spp. cannot be linked to animal contact. For example, in an old study, one third of *Pasteurella* spp. infections were not associated with animal contact, while in a more recent study, in about 20% of patients with bacteremia due to *P. multocida*, no association with animals was noted [56,57].

There are several recent reports of cases of IE by these pathogens. IE is a relatively uncommon disease that is associated with significant morbidity and mortality. The typical causes of IE are Gram-positive bacteria, but Gram-negative bacteria can also be the cause in some instances, as in the case of previous hospitalization or recent exposure to the health care system [58,59,60]. Information regarding any differences in clinical presentation and specific guidelines on managing IE by Gram-negative species is generally inadequate [6,7,60]. Thus, it is important to better to study IE by Gram-negative bacteria in a systematic way to allow for understanding any differences in terms of epidemiology, clinical presentation, treatment and outcomes compared with IE by classic Gram-positive microorganisms. More specifically, IE by *Pasteurella* spp. is a very rare entity with scarce evidence in the literature. This is the first study to systematically review IE by this species to provide data on its characteristics, treatment and outcomes.

The mean age at diagnosis of *Pasteurella* spp. IE herein was 56.4 years, within the reported age range of diagnosis of IE by other non-HACEK Gram-negative microorganisms in the literature (40 to 70 years) [59,61,62,63,64,65,66,67,68,69]. The age at diagnosis of IE in the general cohorts of IE patients was higher, close to 70 years [8,10,11,70]. There were more male patients than female among patients with IE by *Pasteurella* spp., as was also the case with IE by other non-HACEK Gram-negative microorganisms and in other cohorts of patients with IE in the general population [8,9,10,59,61,62,63,64,65,66,67,68,69,70]. A prosthetic valve was present in 21.4% of patients with IE by *Pasteurella* spp., which was a rate similar to that noted in other studies of IE by non-HACEK Gram-negative bacteria (14% to 59%) and similar to the rate noted in cohorts of patients with IE in general [10,11,59,61,62,63,64,65,66,67,68,69,70]. A history of rheumatic fever was present in the medical history of patients diagnosed with IE by *Pasteurella* spp. in 3.6%, similar to the rate in studies of patients with IE in general [11,70]. Liver cirrhosis and immunosuppression are factors identified to be very common among patients with invasive disease by *Pasteurella* spp [71,72]. The association with cirrhosis could in theory be explained by the possibility of altered phagocytic activity and the presence of a portacaval shunt noted in these patients that seemed to play an important role in their developing bacteremia [73,74]. In the present study, 10.7% of patients had cirrhosis, which is a rate similar to that in other studies with IE, while immunosuppression was noted in only 1 patient who had undergone kidney transplantation in the past [11]. However, even though these numbers seem to be relatively low, the small number of the patients included in the present study does not allow for drawing firm conclusions regarding the importance of these factors in any predisposition to IE by *Pasteurella* spp.

The most commonly infected sites in the heart were the aortic valve in 50% and the mitral valve in 30.8% of patients. This is in accordance with other studies with IE by non-HACEK Gram-negative bacteria, where the aortic valve was the most commonly infected valve in 33.3% to 45%, followed by the mitral valve in 26.7% to 40% [63,64,66], or the tricuspid valve in 33% [61]. In other studies, however, the mitral valve was the most commonly infected valve in 31% to 58%, followed by the aortic valve in 17% to 33% [59,65,67,69]. In most studies of IE in the general population, the aortic valve was the most commonly infected valve, followed by the mitral valve [10,70].

The most common symptom regarding clinical presentation was fever, present in 96.3% of patients, while 81.5% of patients developed sepsis, and 21.4% developed septic shock. In other studies with IE by non-HACEK Gram-negative bacteria, fever was present in 90.5% to 100% of patients [59,61,63,64,65,66,67,69], and sepsis was noted in 39% to 84.6% [63,64,65,66,67,69], while septic shock was noted in up to 30% [64,65,68,69]. In patients with IE in general, fever was noted in 84%, and shock was diagnosed in 9% [10,11]. A diagnosis of heart failure occurred with 21.4% of patients with IE by *Pasteurella* spp., which is similar to the rate in cases of non-HACEK Gram-negative IE, which ranged from 8% to 39.3% [59,61,63,64,65,66,67,68,69], and lower than the rate noted in IE in general, which was between 33% and 52% [10,70]. Immunologic phenomena in IE by *Pasteurella* spp. were present in 17.9%, which is a rate similar to the one noted in other studies with IE by non-HACEK Gram-negative bacteria, where that rate ranged from 8% to 27% [59,61,62,63,64,65,66,67,68,69]. Immunologic phenomena more commonly included glomerulonephritis and petechiae. Furthermore, embolic phenomena in IE by *Pasteurella* spp. were present in 14.3%, a rate slightly lower than the one noted in other studies with IE by non-HACEK Gram-negative bacteria, where that rate ranged from 17% to 65% [59,61,62,63,64,65,66,67,68,69]. In patients with IE in general, the corresponding rates for immunologic and embolic phenomena were 15.9% and 15%-45% respectively [10,11]. A paravalvular abscess was diagnosed in 14.8% of patients with IE by *Pasteurella* spp., within the rate noted in other cases of IE by non-HACEK Gram-negative bacteria, 8% to 42% [59,61,63,64,65,66,67,69].

This study identified cephalosporins, aminopenicillins and penicillin as the most common antimicrobials used in the treatment of IE by *Pasteurella* spp. This is in line with the fact that antimicrobial susceptibility to beta-lactams was 100% in studies with available data in the present review. Indeed, beta-lactams are antimicrobials for which minimal antimicrobial resistance has been documented among *Pasteurella* strains. For example, in a study where different strains of *P. multocida* were isolated from animals, antimicrobial resistance to amoxicillin was less than 5% [75]. Similarly, the antimicrobial resistance of *P. multocida* to ampicillin was lower than 10% in other studies with data from animals; however, the antimicrobial resistance of *P. haemolytica* to ampicillin was higher, and was close to 30% [76]. This pattern of antimicrobial susceptibility also holds for other antimicrobials, such as tetracyclines, where antimicrobial resistance was also 0%, while aminoglycosides had an antimicrobial resistance rate of 11.1%, indicating that even in patients with severe penicillin allergy, there are alternative options for antimicrobial therapy.

Herein, overall mortality was 17.9% and was associated directly with the episode of IE. This mortality is close to the rate in other studies of IE by non-HACEK Gram-negative bacteria, where it was as high as 43.8%, and comparable with the rates noted in patients with IE in general, 11–40% [8,9,10,11,59,61,62,63,64,65,66,67,68,70].

*Pastereulla* spp. can cause SSTIs, sepsis, osteomyelitis, septic arthritis, respiratory tract infections and meningitis, and more serious infections with higher mortality are seen in neonates, the elderly and immunocompromised patients [77,78,79,80]. In most cases of SSTI, septic arthritis and osteomyelitis develop after an animal bite. Respiratory tract infections by *Pasteurella* spp. may also occur, involving pharyngitis, sinusitis, otitis media, epiglottitis, bronchitis, pneumonia, lung abscess or empyema [47,78,81,82,83,84]. The majority of these patients have a previous history of a chronic respiratory disease, such as COPD [85]. Among lower respiratory tract infections by *Pasteurella* spp., pneumonia is the most commonly diagnosed infection, concomitant bacteremia is noted in almost half the patients and mortality is seen in almost 30% [47,86,87]. Meningitis by *Pasteurella* spp. is a disease diagnosed in patients at the extremes of age, usually younger than 12 months or older than 60 years [47,88,89]. Bacteremia by *Pasteurella* spp. is more commonly diagnosed in the setting of another infection, usually an SSTI, pneumonia, meningitis or septic arthritis, but it can also be diagnosed without another obvious site of infection [47,90]. Mortality was 31% at 30 days after diagnosis and was more common among patients who had a major comorbidity, such as solid organ transplantation, malignancy, diabetes or significant liver dysfunction [71]. In the present study, immunosuppression was not frequent. Furthermore, the mean age was 56.4 years and relatively few were patients who were older than 70 years. This could partially explain the lower mortality noted in the present review, as immunosuppression and extremes of age are associated with increased mortality in other infections by *Pasteurella* spp.

This systematic review has some notable limitations. Firstly, it consists solely of case reports. Thus, the results should be read with caution as the quality of evidence presented by these studies is very low. Moreover, the possibility of publication bias may have affected the presented data. However, as there is no original study with an adequate number of patients giving information on IE by *Pasteurella* spp., we could not have used another methodology to study these infections. Finally, there is a possibility that some studies may have been missed during the search process, thus affecting the number of the included studies.

## 5. Conclusions

To conclude, this systematic review describes the epidemiology, microbiology, clinical characteristics, treatment and outcomes of IE by *Pasteurella* spp. Cephalosporins, aminopenicillins and penicillin were the antimicrobials that were most commonly used.

## Figures and Tables

**Table 1:** Characteristics of 28 patients with infective endocarditis by *Pasteurella* spp. Values show cases among patients with available data.

Characteristic	Value (*n* Out of 28, Unless Otherwise Mentioned)
Male, *n* (%)	20 (71.4%)
Age, mean (SD) in years	56.4 (15.7)
Predisposing factors	
Animal bite or close contact, *n* (%)	21 (75%)
Prosthetic valve, *n* (%)	6 (21.4%)
Bad teeth hygiene or recent dental work, *n* (%)	3 (10.7%)
Previous IE, *n* (%)	3 (10.7%)
Liver cirrhosis, *n* (%)	3 (10.7%)
IVDU, *n* (%)	2 (7.1%)
Rheumatic fever, *n* (%)	1 (3.6%)
ESRD on hemodialysis, *n* (%)	1 (3.6%)
Immunosuppression, *n* (%)	1 (3.6%)
Recent cardiac surgery (within three months), *n* (%)	0 (0%)
Congenital heart disease, *n* (%)	0 (0%)
Valve localization	
Aortic valve, *n* (%)	13 out of 26 (50%)
Mitral valve, *n* (%)	8 out of 26 (30.8%)
Tricuspid valve, *n* (%)	3 out of 26 (11.5%)
Pulmonary valve, *n* (%)	1 out of 26 (3.8%)
Eustachian valve, *n* (%)	1 out of 26 (3.8%)
Mural endocardium, *n* (%)	1 out of 26 (3.8%)
Multiple valves, *n* (%)	1 out of 26 (3.8%)
Microbiological data	
*Pasteurella multocida*, *n* (%)	20 (71.4%)
*Pasteurella dagmatis*, *n* (%)	3 (10.7%)
*Pasteurellapneumotropica*, *n* (%)	2 (7.1%)
*Pasteurellahaemolytica*, *n* (%)	2 (7.1%)
*Pasteurellaaerogenes*, *n* (%)	1 (3.6%)
Antimicrobial resistance	
Penicillin, *n* (%)	0 out of 11 (0%)
Aminoglycosides, *n* (%)	1 out of 9 (11.1%)
Cephalosporins, *n* (%)	0 out of 8 (0%)
Ampicillin, *n* (%)	0 out of 8 (0%)
Tetracyclines, *n* (%)	0 out of 7 (0%)
Method of diagnosis	
Transthoracic echocardiography, *n* (%)	12 out of 25 (48%)
Transesophageal echocardiography, *n* (%)	7 out of 25 (28%)
Valve culture, *n* (%)	7 out of 25 (28%)
Autopsy, *n* (%)	3 (10.7%)
Empirical diagnosis, *n* (%)	1 (3.6%)
Clinical characteristics	
Fever, *n* (%)	26 out of 27 (96.3%)
Sepsis, *n* (%)	22 out of 27 (81.5%)
Septic shock, *n* (%)	6 (21.4%)
Heart failure, *n* (%)	6 (21.4%)
Immunologic phenomena, *n* (%)	5 (17.9%)
Embolic phenomena, *n* (%)	4 (14.3%)
Paravalvular abscess, *n* (%)	4 out of 27 (14.8%)
Treatment	
Duration of treatment in weeks, median (IQR)	6 (4.5–6.5)
Cephalosporin, *n* (%)	12 out of 27 (44.4%)
Aminopenicillins, *n* (%)	10 out of 27 (37%)
Penicillin, *n* (%)	8 out of 27 (29.6%)
Piperacillin and tazobactam, *n* (%)	5 out of 27 (18.5%)
Aminoglycoside, *n* (%)	6 out of 27 (22.2%)
Quinolone, *n* (%)	5 out of 27 (18.5%)
Carbapenem, *n* (%)	2 out of 27 (7.4%)
Tetracycline, *n* (%)	2 out of 27 (7.4%)
Surgical management, *n* (%)	12 (42.9%)
Outcomes	
Clinical cure, *n* (%)	23 (82.1%)
Deaths due to infection, *n* (%)	5 (17.9%)
Deaths overall, *n* (%)	5 (17.9%)

ESRD: end-stage renal disease; IE: infective endocarditis; IQR: interquartile range; IVDU: intravenous drug use; SD: standard deviation.

**Table 2:** Characteristics of patients with infective endocarditis by *Pasteurella* spp. according to whether they survived or died from the infection. Values show cases among patients with available data.

Characteristic	Patients Who Survived (*n* Out of 23, Unless Otherwise Mentioned)	Patients Who Died (*n* Out of 5, Unless Otherwise Mentioned)
Male, *n* (%)	16 (69.6%)	4 (80%)
Age, mean (SD) in years	55.9 (16.5)	58.8 (12.8)
Predisposing factors		
Animal bite or close contact, *n* (%)	18 (78.3%)	3 (60%)
Prosthetic valve, *n* (%)	6 (26.1%)	0 (0%)
Bad teeth hygiene or recent dental work, *n* (%)	3 (13%)	0 (0%)
Previous IE, *n* (%)	3 (13%)	0 (0%)
Liver cirrhosis, *n* (%)	2 (8.7%)	1 (20%)
IVDU, *n* (%)	2 (8.7%)	0 (0%)
Rheumatic fever, *n* (%)	0 (0%)	1 (20%)
ESRD on hemodialysis, *n* (%)	0 (0%)	1 (20%)
Immunosuppression, *n* (%)	1 (4.3%)	0 (0%)
Valve localization		
Aortic valve, *n* (%)	11 out of 22 (50%)	2 out of 4 (50%)
Mitral valve, *n* (%)	6 out of 22 (27.3%)	2 out of 4 (50%)
Tricuspid valve, *n* (%)	3 out of 22 (13.6%)	0 out of 4 (0%)
Pulmonary valve, *n* (%)	1 out of 22 (4.5%)	0 out of 4 (0%)
Eustachian valve, *n* (%)	1 out of 22 (4.5%)	0 out of 4 (0%)
Mural endocardium, *n* (%)	1 out of 22 (4.5%)	0 out of 4 (0%)
Multiple valves, *n* (%)	1 out of 22 (4.5%)	0 out of 4 (0%)
Microbiological data		
*Pasteurella multocida*, *n* (%)	17 (73.9%)	3 (60%)
*Pasteurella dagmatis*, *n* (%)	3 (13%)	0 (0%)
*Pasteurellapneumotropica*, *n* (%)	2 (8.7%)	0 (0%)
*Pasteurellahaemolytica*, *n* (%)	0 (0)	2 (40%)
*Pasteurellaaerogenes*, *n* (%)	1 (4.3%)	0 (0%)
Method of diagnosis		
Transthoracic echocardiography, *n* (%)	10 out of 20 (50%)	2 (40%)
Transesophageal echocardiography, *n* (%)	7 out of 20 (35%)	0 (0%)
Valve culture, *n* (%)	6 out of 20 (30%)	1 (20%)
Autopsy, *n* (%)	0 (0%)	3 (60%)
Empirical diagnosis, *n* (%)	1 (4.3%)	0 (0%)
Clinical characteristics		
Fever, *n* (%)	21 out of 22 (95.5%)	5 (100%)
Sepsis, *n* (%)	17 out of 22 (77.3%)	5 (100%)
Septic shock, *n* (%)	3 (13%)	3 (60%)
Heart failure, *n* (%)	5 (21.7%)	1 (20%)
Immunologic phenomena, *n* (%)	4 (17.4%)	1 (20%)
Embolic phenomena, *n* (%)	2 (8.7%)	2 (40%)
Paravalvular abscess, *n* (%)	4 out of 22 (18.2%)	0 (0%)
Treatment		
Duration of treatment in weeks, median (IQR)	6 (4.5–6.5)	NA
Cephalosporin, *n* (%)	11 out of 22 (50%)	1 (20%)
Aminopenicillins, *n* (%)	7 out of 22 (31.8%)	3 (60%)
Penicillin, *n* (%)	7 out of 22 (31.8%)	1 (20%)
Piperacillin and tazobactam, *n* (%)	4 out of 22 (18.2%)	1 (20%)
Aminoglycoside, *n* (%)	3 out of 22 (13.6%)	3 (60%)
Quinolone, *n* (%)	4 out of 22 (18.2%)	1 (20%)
Carbapenem, *n* (%)	2 out of 22 (9.1%)	0 (0%)
Tetracycline, *n* (%)	1 out of 22 (4.5%)	1 (20%)
Surgical management, *n* (%)	12 (52.2%)	0 (0%)

ESRD: end-stage renal disease; IE: Infective Endocarditis; IQR: interquartile range; IVDU: intravenous drug use; NA: not applicable; SD: standard deviation.

## Data Availability

The data presented in this study are available on request from the corresponding author.

## Supplementary Materials

The following supporting information can be downloaded at: https://www.mdpi.com/article/10.3390/jcm11175037/s1, Table S1: Characteristics of the included studies.

## References

[1] Bennett J.E., Dolin E., Blaser M.J. (2019). Mandell, Douglas, And Bennett’s Principles and Practice of Infectious Diseases.

[2] Zbinden R., Jorgensen J.H., Carroll K.C., Funke G., Pfaller M.A., Landry M.L., Richter S.S., Warnock D.W. (2015). Aggregatibacter, Capnocytophaga, Eikenella, Kingella, Pasteurella, and Other Fastidious or Rarely Encountered Gram-Negative Rods. Manual of Clinical Microbiology.

[3] Townsend K.M., Boyce J.D., Chung J.Y., Frost A.J., Adler B. (2001). Genetic Organization of *Pasteurella multocida* Cap Loci and Development of a Multiplex Capsular PCR Typing System. J. Clin. Microbiol..

[4] Arumugam N.D., Ajam N., Blackall P.J., Asiah N.M., Ramlan M., Maria J., Yuslan S., Thong K.L. (2011). Capsular Serotyping of *Pasteurella multocida* from Various Animal Hosts—A Comparison of Phenotypic and Genotypic Methods. Trop. Biomed..

[5] Wilson B.A., Ho M. (2013). *Pasteurella multocida*: From Zoonosis to Cellular Microbiology. Clin. Microbiol. Rev..

[6] Baddour L.M., Wilson W.R., Bayer A.S., Fowler V.G., Tleyjeh I.M., Rybak M.J., Barsic B., Lockhart P.B., Gewitz M.H., Levison M.E. (2015). Infective Endocarditis in Adults: Diagnosis, Antimicrobial Therapy, and Management of Complications: A Scientific Statement for Healthcare Professionals From the American Heart Association. Circulation.

[7] Wang A., Gaca J.G., Chu V.H. (2018). Management Considerations in Infective Endocarditis: A Review. JAMA.

[8] Habib G., Erba P.A., Iung B., Donal E., Cosyns B., Laroche C., Popescu B.A., Prendergast B., Tornos P., Sadeghpour A. (2019). Clinical Presentation, Aetiology and Outcome of Infective Endocarditis. Results of the ESC-EORP EURO-ENDO (European Infective Endocarditis) Registry: A Prospective Cohort Study. Eur. Heart J..

[9] Shah A.S.V., McAllister D.A., Gallacher P., Astengo F., Rodríguez Pérez J.A., Hall J., Lee K.K., Bing R., Anand A., Nathwani D. (2020). Incidence, Microbiology, and Outcomes in Patients Hospitalized with Infective Endocarditis. Circulation.

[10] Cresti A., Chiavarelli M., Scalese M., Nencioni C., Valentini S., Guerrini F., D’Aiello I., Picchi A., De Sensi F., Habib G. (2017). Epidemiological and Mortality Trends in Infective Endocarditis, a 17-Year Population-Based Prospective Study. Cardiovasc. Diagn. Ther..

[11] Papakonstantinou P.E., Samonis G., Andrianaki A.M., Christofaki M., Dimopoulou D., Papadakis J., Gikas A., Kofteridis D.P. (2018). Epidemiology, Microbiological and Clinical Features, Treatment, and Outcomes of Infective Endocarditis in Crete, Greece. Infect. Chemother..

[12] Porter R.S., Hay C.M. (2020). Pasteurella Endocarditis: A Case Report and Statistical Analysis of the Literature. Case Rep. Infect. Dis..

[13] Stroup D.F., Berlin J.A., Morton S.C., Olkin I., Williamson G.D., Rennie D., Moher D., Becker B.J., Sipe T.A., Thacker S.B. (2000). Meta-Analysis of Observational Studies in Epidemiology: A Proposal for Reporting. Meta-Analysis Of Observational Studies in Epidemiology (MOOSE) Group. JAMA.

[14] Wallace B.C., Small K., Brodley C.E., Lau J., Trikalinos T.A. Deploying an Interactive Machine Learning System in an Evidence-Based Practice Center: Abstrackr. Proceedings of the 2nd ACM SIGHIT International Health Informatics Symposium.

[15] Li J.S., Sexton D.J., Mick N., Nettles R., Fowler V.G., Ryan T., Bashore T., Corey G.R. (2000). Proposed Modifications to the Duke Criteria for the Diagnosis of Infective Endocarditis. Clin. Infect. Dis..

[16] Guyatt G.H., Oxman A.D., Vist G.E., Kunz R., Falck-Ytter Y., Alonso-Coello P., Schünemann H.J. (2008). GRADE Working Group GRADE: An Emerging Consensus on Rating Quality of Evidence and Strength of Recommendations. BMJ.

[17] Doty G.L., Loomus G.N., Wolf P.L. (1963). Pasteurella Endocarditis. N. Engl. J. Med..

[18] Gump D.W., Holden R.A. (1972). Endocarditis Caused by a New Species of Pasteurella. Ann. Intern. Med..

[19] Lehmann V., Knutsen S.B., Ragnhildstveit E., Skagseth E., Solberg C.O. (1977). Endocarditis Caused by *Pasteurella multocida*. Scand. J. Infect. Dis..

[20] Singh C.P., Spurrell J.R. (1983). *Pasteurella multocida* Endocarditis. Br. Med. J. (Clin. Res. Ed.).

[21] Salmon D., Fantin B., Bricaire F., Vilde J.L., Pangon B., Ferand D. (1989). Endocarditis Due to *Pasteurella multocida* with Glomerulonephritis. Am. J. Med..

[22] Yaneza A.L., Jivan H., Kumari P., Togoo M.S. (1991). Pasteurella Haemolytica Endocarditis. J. Infect..

[23] Hombal S.M., Dincsoy H.P. (1992). *Pasteurella multocida* Endocarditis. Am. J. Clin. Pathol..

[24] Yamamoto K., Ikeda U., Ogawa C., Fukazawa H., Eto M., Shimada K. (1993). Pasteurella Ureae Endocarditis. Intern. Med..

[25] Sorbello A.F., O’Donnell J., Kaiser-Smith J., Fitzharris J., Shinkarow J., Doneson S. (1994). Infective Endocarditis Due to Pasteurella Dagmatis: Case Report and Review. Clin. Infect. Dis..

[26] Genne D., Siegrist H.H., Monnier P., Nobel M., Humair L., de Torrente A. (1996). *Pasteurella multocida* Endocarditis: Report of a Case and Review of the Literature. Scand. J. Infect. Dis..

[27] Nettles R.E., Sexton D.J. (1997). *Pasteurella multocida* Prosthetic Valve Endocarditis: Case Report and Review. Clin. Infect. Dis..

[28] Vasquez J.E., Ferguson D.A., Bin-Sagheer S., Myers J.W., Ramsak A., Wilson M.A., Sarubbi F.A. (1998). *Pasteurella multocida* Endocarditis: A Molecular Epidemiological Study. Clin. Infect. Dis..

[29] Rosenbach K.A., Poblete J., Larkin I. (2001). Prosthetic Valve Endocarditis Caused by Pasteurella Dagmatis. South Med. J..

[30] Fukumoto Y., Moriyama Y., Iguro Y., Toda R., Taira A. (2002). *Pasteurella multocida* Endocarditis: Report of a Case. Surg. Today.

[31] Al-Ghonaim M.A., Abba A.A., Al-Nozha M. (2006). Endocarditis Caused by *Pasteurella multocida*. Ann. Saudi Med..

[32] Graf S., Binder T., Heger M., Apfalter P., Simon N., Winkler S. (2007). Isolated Endocarditis of the Pulmonary Valve Caused by *Pasteurella multocida*. Infection.

[33] Reinsch N., Plicht B., Lind A., Jánosi R.A., Buck T., Kamler M., Jakob H., Naber C.K., Erbel R. (2008). Recurrent Infective Endocarditis with Uncommon Gram-Negative *Pasteurella multocida* and Pseudomonas Aeruginosa: A Case Report. J. Heart Valve Dis..

[34] Naba M.R., Araj G.F., Kanafani Z.A., Kanj S.S. (2009). First Case of *Pasteurella multocida* Endocarditis of the Tricuspid Valve: A Favorable Outcome Following Medical Treatment. Int. J. Infect. Dis..

[35] Strahm C., Goldenberger D., Gutmann M., Kuhnert P., Graber P. (2012). Prosthetic Valve Endocarditis Caused by a Pasteurella Dagmatis-like Isolate Originating from a Patient’s Cat. J. Clin. Microbiol..

[36] Tirmizi A., Butt S., Molitorisz S. (2012). First Reported Case of Pasteurella Pneumotropica Tricuspid Valve Endocarditis. Int. J. Cardiol..

[37] Satta G., Gorton R.L., Kandil H. (2012). Prosthetic Valve Endocarditis Caused by Pasteurella in a Penicillin Allergic Patient: Challenges in Diagnosis and Treatment. Infect. Dis. Rep..

[38] Mikaberidz N., Li E.Y., Taub C.C. (2013). *Pasteurella multocida* Infective Endocarditis in an Immunocompetent Patient Complicated by Rhabdomyolysis and Permanent Hearing Loss. J. Cardiovasc. Dis. Res..

[39] Branch J., Kakutani T., Kuroda S., Shiba Y., Kitagawa I. (2015). *Pasteurella multocida* Infective Endocarditis: A Possible Link with Primary Upper Respiratory Tract Infection. Intern. Med..

[40] Guilbart M., Zogheib E., Hchikat A.H., Kirat K., Ferraz L., Guerin-Robardey A.-M., Trojette F., Moubarak-Daher M., Dupont H. (2015). Fatal Multifocal *Pasteurella multocida* Infection: A Case Report. BMC Res. Notes.

[41] Ahlsson A., Friberg Ö., Källman J. (2016). An Angry Cat Causing *Pasteurella multocida* Endocarditis and Aortic Valve Replacement-A Case Report. Int. J. Surg. Case Rep..

[42] Ghanem H., Martin C., Farrer W., Sivasubramanian G. (2021). Eustachian Valve Endocarditis Due to Pasturella Multocida—A Novel Case. Clin. Infect. Pract..

[43] Hung W.-S., Wu M., Jung S.-M., Chu P.-H. (2021). Infective Endocarditis Caused by Pasteurella Aerogenes Possibly from a Dog. Clin. Infect. Pract..

[44] Wilkie I.W., Harper M., Boyce J.D., Adler B. (2012). *Pasteurella multocida*: Diseases and Pathogenesis. Curr. Top. Microbiol. Immunol..

[45] Muntaner L., Suriñach J.M., Zuñiga D., De Sevilla T.F., Ferrer A. (2008). Respiratory Pasteurellosis: Infection or Colonization?. Scand. J. Infect. Dis..

[46] Guillard T., Martin M., Duval V., Brasme L., David C., Vernet-Garnier V., Lebargy F., de Champs C. (2010). Respiratory Tract Colonization by Pasteurella Pneumotropica in a Patient with an Alpha1-Antitrypsin Deficiency Unexpectedly Well Identified by Automated System Vitek 2. Diagn. Microbiol. Infect. Dis..

[47] Weber D.J., Wolfson J.S., Swartz M.N., Hooper D.C. (1984). *Pasteurella multocida* Infections. Report of 34 Cases and Review of the Literature. Medicine.

[48] Holst E., Rollof J., Larsson L., Nielsen J.P. (1992). Characterization and Distribution of *Pasteurella* Species Recovered from Infected Humans. J. Clin. Microbiol..

[49] Abrahamian F.M., Goldstein E.J.C. (2011). Microbiology of Animal Bite Wound Infections. Clin. Microbiol. Rev..

[50] Brook I. (1987). Microbiology of Human and Animal Bite Wounds in Children. Pediatr. Infect. Dis. J..

[51] Francis D.P., Holmes M.A., Brandon G. (1975). *Pasteurella multocida*. Infections after Domestic Animal Bites and Scratches. JAMA.

[52] Dendle C., Looke D. (2008). Review Article: Animal Bites: An Update for Management with a Focus on Infections. Emerg. Med. Australas..

[53] Talan D.A., Citron D.M., Abrahamian F.M., Moran G.J., Goldstein E.J. (1999). Bacteriologic Analysis of Infected Dog and Cat Bites. Emergency Medicine Animal Bite Infection Study Group. N. Engl. J. Med..

[54] Westling K., Farra A., Cars B., Ekblom A.G., Sandstedt K., Settergren B., Wretlind B., Jorup C. (2006). Cat Bite Wound Infections: A Prospective Clinical and Microbiological Study at Three Emergency Wards in Stockholm, Sweden. J. Infect..

[55] Boyanton B.L., Freij B.J., Robinson-Dunn B., Makin J., Runge J.K., Luna R.A. (2016). Neonatal *Pasteurella multocida* Subsp. Septica Meningitis Traced to Household Cats: Molecular Linkage Analysis Using Repetitive-Sequence-Based PCR. J. Clin. Microbiol..

[56] Hubbert W.T., Rosen M.N. (1970). *Pasteurella multocida* Infections. II. Pasteurella multocida Infection in Man Unrelated to Animal Bite. Am. J. Public Health Nations Health.

[57] Vondra M.S., Myers J.P. (2011). *Pasteurella multocida* Bacteremia: Report of 12 Cases in the 21st Century and Comprehensive Review of the Adult Literature. Infect. Dis. Clin. Pract..

[58] Cahill T.J., Prendergast B.D. (2016). Infective Endocarditis. Lancet.

[59] Morpeth S., Murdoch D., Cabell C.H., Karchmer A.W., Pappas P., Levine D., Nacinovich F., Tattevin P., Fernández-Hidalgo N., Dickerman S. (2007). Non-HACEK Gram-Negative Bacillus Endocarditis. Ann. Intern. Med..

[60] Bouza E., Muñoz P., Burillo A. (2021). Gram-Negative Endocarditis: Disease Presentation, Diagnosis and Treatment. Curr. Opin. Infect. Dis..

[61] Loubet P., Lescure F.-X., Lepage L., Kirsch M., Armand-Lefevre L., Bouadma L., Lariven S., Duval X., Yazdanpanah Y., Joly V. (2015). Endocarditis Due to Gram-Negative Bacilli at a French Teaching Hospital over a 6-Year Period: Clinical Characteristics and Outcome. Infect. Dis..

[62] Veve M.P., McCurry E.D., Cooksey G.E., Shorman M.A. (2020). Epidemiology and Outcomes of Non-HACEK Infective Endocarditis in the Southeast United States. PLoS ONE.

[63] Ioannou P., Vougiouklakis G. (2020). Infective Endocarditis by *Proteus* Species: A Systematic Review. Germs.

[64] Ioannou P., Mavrikaki V., Kofteridis D.P. (2021). Infective Endocarditis by *Acinetobacter* Species: A Systematic Review. J. Chemother..

[65] Ioannou P., Vamvoukaki R., Kofteridis D.P. (2021). Infective Endocarditis by *Enterobacter cloacae*: A Systematic Review and Meta-Analysis. J. Chemother..

[66] Ioannou P., Miliara E., Baliou S., Kofteridis D.P. (2021). Infective Endocarditis by *Klebsiella* Species: A Systematic Review. J. Chemother..

[67] Ioannou P., Vougiouklakis G., Baliou S., Miliara E., Kofteridis D.P. (2021). Infective Endocarditis by *Yersinia* Species: A Systematic Review. Trop. Med. Infect. Dis..

[68] Falcone M., Tiseo G., Durante-Mangoni E., Ravasio V., Barbaro F., Ursi M.P., Pasticci M.B., Bassetti M., Grossi P., Venditti M. (2018). Risk Factors and Outcomes of Endocarditis Due to Non-HACEK Gram-Negative Bacilli: Data from the Prospective Multicenter Italian Endocarditis Study Cohort. Antimicrob. Agents Chemother..

[69] Ioannou P., Alexakis K., Spentzouri D., Kofteridis D.P. (2022). Infective Endocarditis by *Serratia* Species: A Systematic Review. J. Chemother..

[70] Giannitsioti E., Skiadas I., Antoniadou A., Tsiodras S., Kanavos K., Triantafyllidi H., Giamarellou H., Hellenic Endocarditis Study Group Nosocomial vs. (2007). Community-Acquired Infective Endocarditis in Greece: Changing Epidemiological Profile and Mortality Risk. Clin. Microbiol. Infect..

[71] Chatelier E., Mahieu R., Hamel J.-F., Chenouard R., Lozac’h P., Sallé A., Kouatchet A., Martin L., Lavigne C., Pailhoriès H. (2020). Pasteurella Bacteraemia: Impact of Comorbidities on Outcome, Based on a Case Series and Literature Review. Int. J. Infect. Dis..

[72] Vesza Z., Boattini M., Pinto M., Marques da Silva P. (2017). *Pasteurella* Infections in a Tertiary Centre—From Cellulitis to Multiple-Organ Failure: Retrospective Case Series. SAGE Open Med. Case Rep..

[73] Orsini J., Perez R., Llosa A., Araguez N. (2013). Non-Zoonotic *Pasteurella multocida* Infection as a Cause of Septic Shock in a Patient with Liver Cirrhosis: A Case Report and Review of the Literature. J. Glob. Infect. Dis..

[74] Alexopoulou A., Agiasotelli D., Vasilieva L.E., Dourakis S.P. (2017). Bacterial Translocation Markers in Liver Cirrhosis. Ann. Gastroenterol..

[75] Bourély C., Cazeau G., Jouy E., Haenni M., Madec J.-Y., Jarrige N., Leblond A., Gay E. (2019). Antimicrobial Resistance of *Pasteurella multocida* Isolated from Diseased Food-Producing Animals and Pets. Vet. Microbiol..

[76] Kehrenberg C., Schulze-Tanzil G., Martel J.L., Chaslus-Dancla E., Schwarz S. (2001). Antimicrobial Resistance in Pasteurella and Mannheimia: Epidemiology and Genetic Basis. Vet. Res..

[77] Nakwan N., Nakwan N., Atta T., Chokephaibulkit K. (2009). Neonatal Pasteurellosis: A Review of Reported Cases. Arch. Dis. Child. Fetal Neonatal. Ed..

[78] Giordano A., Dincman T., Clyburn B.E., Steed L.L., Rockey D.C. (2015). Clinical Features and Outcomes of *Pasteurella multocida* Infection. Medicine.

[79] Rhea S., Weber D.J., Poole C., Cairns C. (2014). Risk Factors for Hospitalization after Dog Bite Injury: A Case-Cohort Study of Emergency Department Visits. Acad Emerg. Med..

[80] Christenson E.S., Ahmed H.M., Durand C.M. (2015). *Pasteurella multocida* Infection in Solid Organ Transplantation. Lancet Infect. Dis..

[81] Klein N.C., Cunha B.A. (1997). *Pasteurella multocida* Pneumonia. Semin. Respir. Infect..

[82] Byrd R.P., Roy T.M. (2003). *Pasteurella multocida* Respiratory Infection: An Important Cat-Associated Zoonosis. Arch. Intern. Med..

[83] Marinella M.A. (2004). Community-Acquired Pneumonia Due to *Pasteurella multocida*. Respir. Care.

[84] Harris P.J., Osswald M.B. (2010). *Pasteurella multocida* Epiglottitis: A Review and Report of a New Case with Associated Chronic Lymphocytic Leukemia. Ear. Nose Throat J..

[85] Seki M., Sakata T., Toyokawa M., Nishi I., Tomono K. (2016). A Chronic Respiratory *Pasteurella multocida* Infection Is Well-Controlled by Long-Term Macrolide Therapy. Intern. Med..

[86] Kopita J.M., Handshoe D., Kussin P.S., Kelemen M. (1993). Cat Germs! Pleuropulmonary Pasteurella Infection in an Old Man. North Carol. Med. J..

[87] Ferreira J., Treger K., Busey K. (2015). Pneumonia and Disseminated Bacteremia with *Pasteurella multocida* in the Immune Competent Host: A Case Report and a Review of the Literature. Respir. Med. Case Rep..

[88] Green B.T., Ramsey K.M., Nolan P.E. (2002). *Pasteurella multocida* Meningitis: Case Report and Review of the Last 11 y. Scand. J. Infect. Dis..

[89] O’Neill E., Moloney A., Hickey M. (2005). *Pasteurella multocida* Meningitis: Case Report and Review of the Literature. J. Infect..

[90] Kimura R., Hayashi Y., Takeuchi T., Shimizu M., Iwata M., Tanahashi J., Ito M. (2004). *Pasteurella multocida* Septicemia Caused by Close Contact with a Domestic Cat: Case Report and Literature Review. J. Infect. Chemother..

