# Supplementary material for: Infective Endocarditis by Pasteurella Species: A Systematic Review
Source: J Clin Med. 2022 Aug 27;11(17):5037. doi: 10.3390/jcm11175037 (PMC9456824; doi:10.3390/jcm11175037)
Supplement: Supplementary file 1 [file jcm-11-05037-s001.zip › jcm-1835922-supplementary.pdf]

Supplementary Table S1. Characteristics of the included studies.

Study, year published	Number of patients	Age (years)	Gender	Site of infection, n (%)	Microbiology of infection, n (%)	Treatment administered	Infection Outcomes, n (%)
Doty et al, 1963 [17]	1	54	Female	MV 1 (100)	*P. haemolytica* 1 (100)	Penicillin 1 (100)<br>Aminoglycoside 1 (100)	Clinical cure[a] 0 (0)<br>Death 1 (100)<br>Overall mortality 1 (100)
Gump et al, 1972 [18]	1	44	Male	NR 1 (100)	*P. multocida* 1 (100)	Penicillin 1 (100)	Clinical cure 1 (100)<br>Overall mortality 0 (0)
Lehmann et al, 1977 [19]	1	51	Male	AoV 1 (100)	*P. multocida* 1 (100)	Penicillin 1 (100)<br>Surgical management 1 (100)	Clinical cure 1 (100)<br>Overall mortality 0 (0)
Singh et al, 1983 [20]	1	50	Male	MV 1 (100)	*P. multocida* 1 (100)	Penicillin 1 (100)<br>Aminoglycoside 1 (100)	Clinical cure 1 (100)<br>Overall mortality 0 (0)
Salmon et al, 1989 [21]	1	63	Female	AoV 1 (100)	*P. multocida* 1 (100)	Cephalosporin 1 (100)<br>Surgical management 1 (100)	Clinical cure 1 (100)<br>Overall mortality 0 (0)
Yaneza et al, 1990 [22]	1	40	Male	AoV 1 (100)	*P. haemolytica* 1 (100)	Aminopenicillin 1 (100)<br>Cephalosporin 1 (100)	Clinical cure 0 (0)<br>Death 1 (100)<br>Overall mortality 1 (100)
Hombal et al, 1992 [23]	1	61	Male	AoV 1 (100)	*P. multocida* 1 (100)	Aminoglycoside 1 (100)	Clinical cure 0 (0)<br>Death 1 (100)<br>Overall mortality 1 (100)
Yamamoto et al, 1993 [24]	1	59	Male	MV 1 (100)	*P. dagmatis* 1 (100)	Aminopenicillin 1 (100)<br>Cephalosporin 1 (100)	Clinical cure 1 (100)<br>Overall mortality 0 (0)
Sorbello et al, 1994 [25]	1	55	Female	MV 1 (100)	*P. pneumotropica* 1 (100)	Cephalosporin 1 (100)	Clinical cure 1 (100)<br>Overall mortality 0 (0)
Genne et al, 1996 [26]	1	38	Female	AoV 1 (100)	*P. multocida* 1 (100)	Penicillin 1 (100)<br>Cephalosporin 1 (100)	Clinical cure 1 (100)<br>Overall mortality 0 (0)
Nettles et al, 1997 [27]	1	72	Female	AoV 1 (100)	*P. multocida* 1 (100)	Aminopenicillin 1 (100)	Clinical cure 1 (100)<br>Overall mortality 0 (0)
Vasquez et al, 1998 [28]	1	65	Male	NR 1 (100)	*P. multocida* 1 (100)	Aminopenicillin 1 (100)	Clinical cure 0 (0)<br>Death 1 (100)<br>Overall mortality 1 (100)
Rosenbach et al, 2001	1	78	Male	AoV 1 (100)	*P. dagmatis* 1 (100)	Cephalosporin 1 (100)	Clinical cure 1 (100)

[29]						Aminoglycoside 1 (100)	Overall mortality 0 (0)
Fukumoto et al, 2002 [30]	1	48	Male	MV 1 (100)	*P. multocida* 1 (100)	Penicillin 1 (100)<br>Cephalosporin 1 (100)<br>Surgical management 1 (100)	Clinical cure 1 (100)<br>Overall mortality 0 (0)
Al Ghonaim et al, 2005 [31]	1	50	Male	AoV 1 (100)	*P. multocida* 1 (100)	Penicillin 1 (100)<br>Aminoglycoside 1 (100)<br>Surgical management 1 (100)	Clinical cure 1 (100)<br>Overall mortality 0 (0)
Graf et al, 2007 [32]	1	36	Male	PV 1 (100)	*P. multocida* 1 (100)	Aminopenicillin 1 (100)<br>Surgical management 1 (100)	Clinical cure 1 (100)<br>Overall mortality 0 (0)
Reinsch et al, 2008 [33]	1	66	Male	AoV 1 (100)	*P. multocida* 1 (100)	Aminopenicillin 1 (100)<br>Surgical management 1 (100)	Clinical cure 1 (100)<br>Overall mortality 0 (0)
Naba et al, 2009 [34]	1	88	Female	TrV 1 (100)	*P. multocida* 1 (100)	Aminopenicillin 1 (100)<br>Quinolone 1 (100)	Clinical cure 1 (100)<br>Overall mortality 0 (0)
Strahm et al, 2012 [35]	1	77	Male	AoV 1 (100)	*P. dagmatis* 1 (100)	Penicillin 1 (100)<br>Cephalosporin 1 (100)<br>Surgical management 1 (100)	Clinical cure 1 (100)<br>Overall mortality 0 (0)
Tirmizi et al, 2012 [36]	1	34	Male	TrV 1 (100)	*P. pneumotropica* 1 (100)	Piperacillin/tazobactam 1 (100)<br>Carbapenem 1 (100)<br>Quinolone 1 (100)	Clinical cure 1 (100)<br>Overall mortality 0 (0)
Satta et al, 2012 [37]	1	38	Male	Mural endocardium 1 (100)	*P. multocida* 1 (100)	Cephalosporin 1 (100)<br>Carbapenem 1 (100)	Clinical cure 1 (100)<br>Overall mortality 0 (0)
Mikaberidz et al, 2013 [38]	1	60	Female	AoV 1 (100)	*P. multocida* 1 (100)	NR 1 (100)<br>Surgical management 1 (100)	Clinical cure 1 (100)<br>Overall mortality 0 (0)
Branch et al, 2015 [39]	1	50	Male	MV 1 (100)	*P. multocida* 1 (100)	Cephalosporin 1 (100)<br>Surgical management 1 (100)	Clinical cure 1 (100)<br>Overall mortality 0 (0)
Guilbart et al, 2015 [40]	1	74	Male	MV 1 (100)	*P. multocida* 1 (100)	Aminopenicillin 1 (100)<br>Piperacillin/tazobactam 1 (100)<br>Quinolone 1 (100)<br>Aminoglycoside 1 (100)<br>Tetracycline 1 (100)	Clinical cure 0 (0)<br>Death 1 (100)<br>Overall mortality 1 (100)

Ahlsson et al, 2016 [41]	1	70	Male	AoV 1 (100)	*P. multocida* 1 (100)	Aminopenicillin 1 (100)<br>Piperacillin/tazobactam 1 (100)<br>Cephalosporin 1 (100)<br>Quinolone 1 (100)<br>Surgical management 1 (100)	Clinical cure 1 (100)<br>Overall mortality 0 (0)
Porter et al, 2020 [12]	1	66	Female	AoV 1 (100)	*P. multocida* 1 (100)	Piperacillin/tazobactam 1 (100)<br>Quinolone 1 (100)<br>Tetracycline 1 (100)<br>Surgical management 1 (100)	Clinical cure 1 (100)<br>Overall mortality 0 (0)
Ghanem et al, 2021 [42]	1	71	Male	EV 1 (100)	*P. multocida* 1 (100)	Aminopenicillin 1 (100)<br>Piperacillin/tazobactam 1 (100)	Clinical cure 1 (100)<br>Overall mortality 0 (0)
Hung et al, 2021 [43]	1	21	Male	MV 1 (100)	*P. aerogenes* 1 (100)	Cephalosporin 1 (100)<br>Surgical management 1 (100)	Clinical cure 1 (100)<br>Overall mortality 0 (0)

[a] Defined as clinical resolution of the infection as a result of treatment; AoV: aortic valve; EV: Eustachian valve; MV: mitral valve; NR: not reported; PV: pulmonary valve; TrV: tricuspid valve
